# A Case of Multifocal Ectopic Purkinje-related Premature Contractions…While Pregnant

**DOI:** 10.19102/icrm.2024.15031

**Published:** 2024-03-15

**Authors:** Connor Oates, Elizabeth Bierbower, Susan O’Donoghue

**Affiliations:** 1 Georgetown University-Washington Hospital Center, Washington, DC, USA

**Keywords:** Purkinje-related premature contractions, pregnancy, *SCN5A*

## Abstract

Multifocal ectopic Purkinje-related premature contractions are a unique electrophysiological finding that can be characteristic of a rare sodium channelopathy. We describe the medical management of this rare channelopathy in a patient who was pregnant.

## Case presentation

A 25-year-old, 21-week gravid woman with a history of heart failure with reduced ejection fraction due to suspected myocarditis and typical atrial flutter status post-ablation of the cavotricuspid isthmus (2021) presented to the hospital from her high-risk obstetrician’s office with symptoms of palpitations.

**Figure 1:**
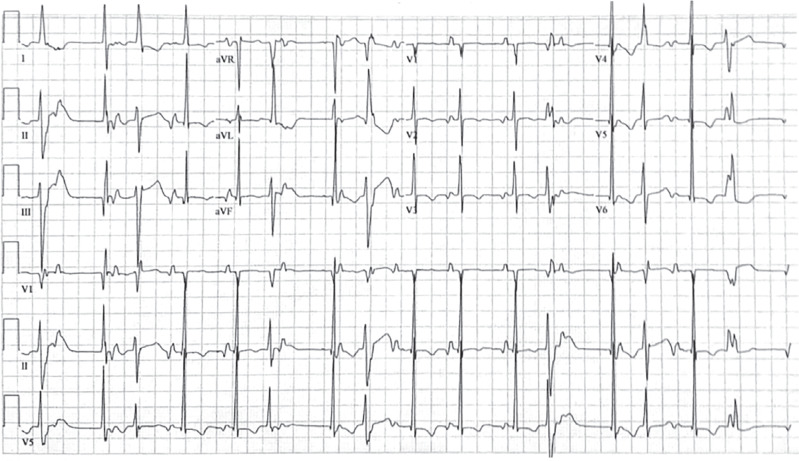
Baseline electrocardiogram demonstrating ectopic atrial rhythm, multifocal junctional beats with right and left bundle branch morphologies regularly followed by retrograde atrial conduction, and a left bundle branch morphology premature ventricular contraction with a superior axis.

The baseline electrocardiogram (ECG) **(Figure 1)** demonstrated chaotic electrical activity. There was a dominant ectopic atrial rhythm with rates of ∼80 bpm, multifocal narrow QRS complexes with right and left bundle branch morphologies regularly followed by retrograde atrial conduction, and a left bundle branch morphology premature ventricular contraction with a superior axis. Telemetry monitoring demonstrated that the patient had only rare sinus beats.

**Figure 2:**
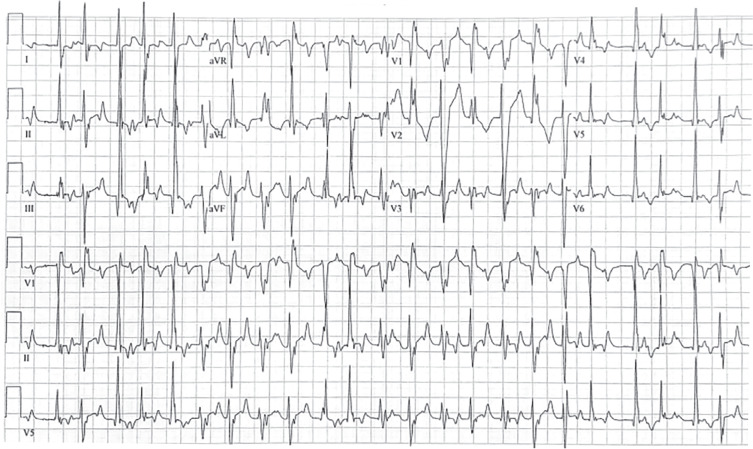
Twelve-lead electrocardiogram demonstrating clinical, symptomatic tachycardia.

**Figure 3:**
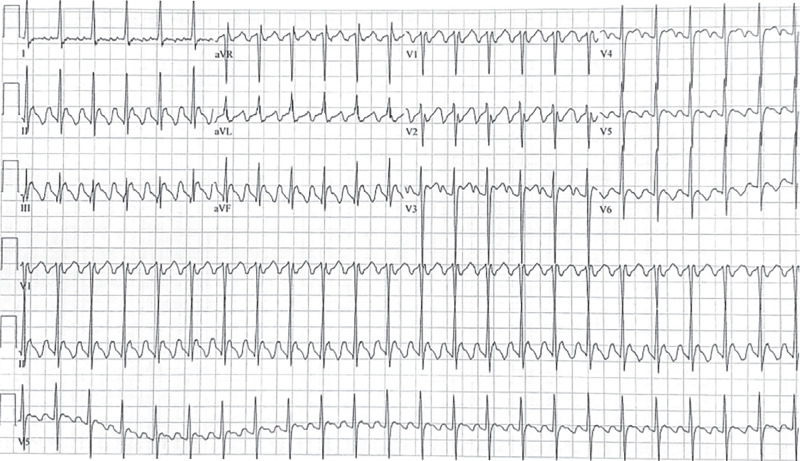
Historical 12-lead electrocardiogram demonstrating when the patient was in typical atrial flutter prior to undergoing catheter ablation.

While on telemetry monitoring, the patient was found to have a clinical tachycardia with recorded rates in the range of 140–160 bpm. Her ECG at the time **(Figure 2)** demonstrated rapid, variable rates of multifocal narrow QRS complexes with right and left bundle branch morphologies regularly followed by retrograde atrial conduction. At times, the junctional beats alternated between right and left bundle branch morphologies. We were also able to collect an ECG demonstrating her atrial flutter prior to undergoing catheter ablation, which was particularly useful for defining a QRS morphology of antegrade atrial–ventricular conduction **(Figure 3)**.

Using transthoracic echocardiography, she was found to have a moderately dilated left ventricle with a left ventricular ejection fraction of 30%, a normal-sized right ventricle with mildly reduced systolic function, and mild biatrial enlargement. The patient’s left ventricular systolic function had not changed since 2020 while on guideline-directed medical therapy. Cardiac magnetic resonance imaging did not identify any evidence of myocardial scar, fibrosis, or infiltration. Ten days of rhythm monitoring demonstrated 12% premature atrial contractions, 10% premature ventricular contractions, and multiple runs of non-sustained ventricular tachycardia. The patient had no known family history of cardiomyopathies, sudden death, or drowning on her mother’s side; however, she was not aware of her father’s family history. Genetic testing demonstrated that the patient had an autosomal dominant c.665G>A (R222Q) gain-of-function mutation in the *SCN5A* gene. Amniocentesis demonstrated that the patient’s fetus did not inherit the c.665G>A (R222Q) variant.

## Discussion

The *SCN5A* gene encodes the pore-forming type 5 subunit α of the voltage-gated sodium channel (Na_V_1.5). Gain-of-function and loss-of-function mutations in this gene have been independently associated with type 3 long QT syndrome, Brugada syndrome, conduction disorders, atrial arrhythmias, and dilated cardiomyopathies. In silico modeling has demonstrated that the c.665G>A gain-of-function mutation causes incomplete repolarization preferentially in Purkinje fibers, which can lead to sporadic early and delayed afterdepolarizations. It is unclear what proportion of cardiomyopathies in this patient population are arrhythmia-induced; however, multiple cases of the normalization of left ventricular systolic function after significant reduction in the burden of ectopy have been reported.

Our patient was in the second trimester of a desired pregnancy, which made her anti-arrhythmic care challenging. Catheter ablation is of limited utility in this patient population. Flecainide, sotalol, and quinidine were anti-arrhythmic drug options considered to have acceptable safety profiles for the mother and baby. Ex vivo data have demonstrated normalization of c.665G>A model myocyte action potentials after treatment with quinidine and flecainide. Similarly, case reports have also described decreases in clinical ectopy after treatment with both quinidine and flecainide. The patient was treated with 324 mg of quinidine gluconate three times daily. Quinidine use during pregnancy has been associated with fetal thrombocytopenia and prolongation of QT; however, quinidine use during pregnancy also has a long, well-described safety record.

The decision was also made to offer the patient implantation of a subcutaneous defibrillator for the primary prevention of sudden cardiac death. It was unclear whether decreased arrhythmic burden would result in an improved left ventricular function, and we believe that cardiac imaging is an imperfect tool for the assessment of the risk for arrhythmic death in patients with this channelopathy. After consulting the obstetrics team, it was felt that the second trimester was the safest time during pregnancy to pursue elective implantation of a defibrillator. The patient decided to return for elective defibrillator implantation, and she was fit for a wearable cardioverter-defibrillator for the interval time.

## Conclusion

Multifocal ectopic Purkinje-related premature ventricular contractions are associated with *SCN5A*-related dilated cardiomyopathy. Treatment with class I anti-arrhythmic agents, such as quinidine, can be an effective treatment strategy for decreasing ectopy in this patient population.

